# Automatic noninvasive measurement of systolic blood pressure using photoplethysmography

**DOI:** 10.1186/1475-925X-8-28

**Published:** 2009-10-26

**Authors:** Meir Nitzan, Amikam Patron, Zehava Glik, Abraham T Weiss

**Affiliations:** 1 Department of Physics/Medical Engineering, Jerusalem College of Technology, Jerusalem, Israel; 2 Department of Geriatrics, Barzilay Medical Center, Ashkelon, Israel; 3 Department of Cardiology, Hadassah Medical Center, Jerusalem, Israel

## Abstract

**Background:**

Automatic measurement of arterial blood pressure is important, but the available commercial automatic blood pressure meters, mostly based on oscillometry, are of low accuracy.

**Methods:**

In this study, we present a cuff-based technique for automatic measurement of systolic blood pressure, based on photoplethysmographic signals measured simultaneously in fingers of both hands. After inflating the pressure cuff to a level above systolic blood pressure in a relatively slow rate, it is slowly deflated. The cuff pressure for which the photoplethysmographic signal reappeared during the deflation of the pressure-cuff was taken as the systolic blood pressure. The algorithm for the detection of the photoplethysmographic signal involves: (1) determination of the time-segments in which the photoplethysmographic signal distal to the cuff is expected to appear, utilizing the photoplethysmographic signal in the free hand, and (2) discrimination between random fluctuations and photoplethysmographic pattern. The detected pulses in the time-segments were identified as photoplethysmographic pulses if they met two criteria, based on the pulse waveform and on the correlation between the signal in each segment and the signal in the two neighboring segments.

**Results:**

Comparison of the photoplethysmographic-based automatic technique to sphygmomanometry, the reference standard, shows that the standard deviation of their differences was 3.7 mmHg. For subjects with systolic blood pressure above 130 mmHg the standard deviation was even lower, 2.9 mmHg. These values are much lower than the 8 mmHg value imposed by AAMI standard for automatic blood pressure meters.

**Conclusion:**

The photoplethysmographic-based technique for automatic measurement of systolic blood pressure, and the algorithm which was presented in this study, seems to be accurate.

## Background

The assessment of the systolic and the diastolic arterial blood pressure has both physiological and clinical significance, and several noninvasive methods have been developed for their measurement. Sphygmomanometry, which is based on an external cuff and audible detection of Korotkoff sounds, is considered to be the most accurate non-invasive method, and has been accepted as the reference standard to which other methods are compared. However, sphygmomanometry is not appropriate for use at home and is also prone to several sources of error. These include insufficient hearing acuity of the examiner and the effect on blood pressure by the presence of a physician [1,2]. Such errors are avoided by automatic measurement of the blood pressure, but the available automatic noninvasive blood pressure (NIBP) meters have other sources of error, significantly reducing their accuracy.

Oscillometry, which is the most widely used technique for automatic NIBP measurement, is based on the analysis of the cardiac induced air-pressure oscillations in the pressure-cuff during cuff deflation after inflating it to an air pressure at a level above the systolic blood pressure (SBP). The SBP is determined as the cuff pressure of maximal slope of the oscillometric curve envelope, or the cuff pressure in which the oscillation amplitude is equal to empirically determined fraction (0.4-0.75) of the maximal amplitude [3-5]. (Similar criteria have been developed for the determination of the diastolic blood pressure.) These empirical criteria are the main source of error in oscillometry since they depend on the character of the cuff and on arterial rigidity [4,5].

The low accuracy of the available automatic NIBP meters in the measurement of SBP and diastolic blood pressure can be deduced from the standards of automatic NIBP meters imposed by the Association for the Advancement of Medical Instrumentation (AAMI) and the British Hypertension Society (BHS). Both standards are based on comparing the blood pressure measurements by an automated NIBP meter to those by a sphygmomanometer - the reference standard - performed on 85 subjects [6,7], each examined three times. (The Working Group on Blood Pressure Monitoring of the European Society of Hypertension suggested reducing the number of the examined subjects to 33 [8]). The AAMI standards require that the mean and standard deviation (SD) of the difference between the SBP (or DBP) values measured by the sphygmomanometer and the device under examination do not exceed 5 and 8 mmHg, respectively. According to these standards, a device of SD of 8 mmHg is acceptable even though 37% of its examinations are expected to differ from the reference sphygmomanometer by 8 mmHg (1SD) or more and 5% of its examinations differ from the reference device by 16 mmHg (2SD) or more (assuming normal distribution). Similar demands were required by the BHS. The reason for the acceptance of devices of such a low accuracy is that oscillometry, the most widespread method, is incapable of providing high accuracy measurements.

### Systolic blood pressure measurement

For cuff pressure above SBP value the pressure pulses in the arteries distal to the cuff disappear due to the collapse of the artery under the cuff. This effect can be used for the SBP measurement using Doppler ultrasound or photoplethysmography (PPG) for the detection of the distal pressure pulses [9-13]. Doppler ultrasound measures the oscillations in blood velocity in the conduit arteries and PPG measures the oscillations in tissue blood volume, which are induced by the blood pressure pulses. When the cuff-pressure increases to above SBP the Doppler or PPG pulses disappear, and when the cuff-pressure decreases below SBP these pulses reappear. Hence, the SBP can be determined from the value of the air-pressure in the cuff for which the Doppler or PPG pulses reappear during cuff deflation. These techniques enable the measurement of SBP with no need for empirical formula.

PPG is suitable for blood pressure measurements because of its simplicity and its high signal-to-noise ratio. Despite the high signal-to-noise ratio, the automatic detection of the first PPG pulses immediately after the decrease of the air pressure below the SBP value is not simple. These pulses are often small, making it difficult to identify them reliably from background noise. In the current study, an automated method for the detection of these small PPG pulses was developed and evaluated by comparing the resulting values of the systolic blood pressure to those obtained by sphygmomanometry, the reference standard for blood pressure measurements.

## Methods

### Subjects and Measurement Technique

SBP was measured on 62 male subjects aged 25-75 years, without known cardiovascular disease except hypertension. In previous studies we have found that the PPG signal in female subjects is generally small relative to that of male subjects, and that male subjects younger than 25 years generally have relatively high notch in their PPG signal, which interferes with the automatic analysis for the detection of the PPG pulse. The subjects were seated during the examination, with their hands comfortably laid on the table and they were asked not to move during the examination. All subjects had an arm circumference between 25-35 cm, and a standard pressure-cuff was used in the study. After a rest period of 10 minutes SBP was measured simultaneously by sphygmomanometry (SBP_S_), oscillometry (SBP_OS_) and by means of PPG (SBP_P_) using the same pressure-cuff on the right arm. Each subject was measured three times, as suggested by AAMI and BHS procedures [6,7]. The study was approved by the institutional ethical committee of Hadassah Medical Center, Jerusalem.

The pressure-cuff and the inflation and deflation were common to the three methods for SBP measurement. Using an electronically controlled mechanical pump the air pressure in the cuff was raised to 20 mmHg above SBP value (details provided later) at a rate of about 15 mmHg/s (see below explanation for this slow rate), then gradually reduced at a rate of 2-3 mmHg/s. The air-pressure in the cuff was continuously measured by a piezoelectric transducer (which was calibrated by a mercury manometer) for the oscillometry and PPG method, and by mercury manometer, for the audible sphygmomanometry. SBP by the latter technique was obtained by two trained observers who determined SBP simultaneously and independently by sphygmomanometry, using a double stethoscope. SBP was taken as the mean value of the readings of the two observers.

SBP was also obtained by oscillometry, using the air-pressure oscillations in the cuff, which were continuously measured by a piezoelectric transducer. The SBP was determined as the cuff air-pressure for which the oscillations (at cuff-pressure above that of maximal oscillations) were 0.6 of the maximal oscillations. This experimental value was used since in former examinations it provided us the lowest standard-deviation for the blood-pressure difference between oscillometry and sphygmomanometry.

The PPG signals were recorded from the two index fingers, using the infrared light-sources and the photodetectors of two pulse-oximeter probes (Oxisensor N25, Nelcor). A band-pass filter (0.8-40 Hz) was used in order to reduce slow trend and high frequency noise and the PPG pulse was inverted, so that upward signal was associated with higher blood volume. The PPG signal and the cuff air-pressure data were sampled (250 Hz, 16 bit) by means of an analog-to-digital converter, whose samples were processed by a digital signal processor.

An important component of the technique was the rate at which the cuff was inflated. In preliminary examinations we have found that in some subjects no pulses in the light transmission curve were actually detected until the pressure had decreased significantly below the SBP value, as measured by Korotkoff sounds. This phenomenon was probably due to the mechanical pressure applied by the PPG sensor itself on the finger arteries underneath. When the cuff air-pressure is above SBP, the arteries distal to the cuff drain into the veins so that the arterial blood pressure becomes relatively low [13]. In some cases the arteries under the sensor may collapse under the small pressure exerted by the sensor, and when the cuff air-pressure is just below SBP value, the small blood-volume pulses entering the arteries distal to the cuff, may not be sufficient to open them.

In order to solve the problem, the cuff air-pressure was increased at a relatively low rate, about 15 mmHg/s, in order to increase venous blood volume and pressure by closing the veins under the cuff for significant time before the arterial closure. This technique reduces the blood drainage of the arteries, allowing the arteries under the PPG sensor to remain open and enabling the detection of the PPG pulses even at pressures just below the SBP.

The technique for the measurement of SBP by the PPG signal requires the increase of the cuff air-pressure to a value above SBP, in order to close the artery under the cuff. We used the cessation of the PPG pulses when the cuff air-pressure is above SBP for stopping the inflation when the air-pressure reached the value of 20 mmHg above SBP. The mean initial maximal derivative and mean cardiac period were derived from the PPG pulses before the start of the cuff inflation. After the start of the inflation, for each PPG pulse, the value of the maximal derivative in the neighborhood of the expected time of the next pulse was computed on-line. The PPG pulses were considered to disappear if the value of the maximal derivative was lower than 1% of the mean initial maximal derivative.

### Data analysis

**Figure 1:**
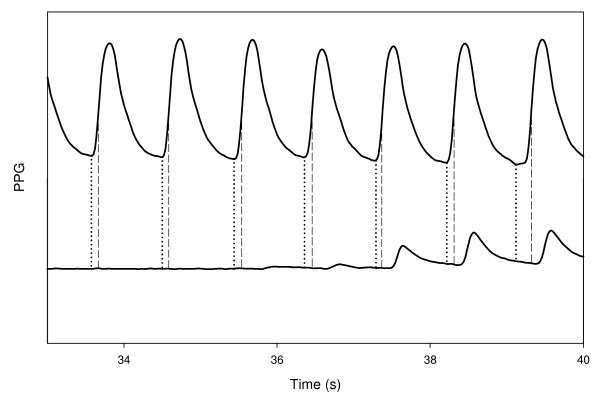
**The PPG signals, in the finger distal to the cuff (below) and in the contralateral finger (above) as a function of the time, during the deflation period, when cuff pressure decreases to below SBP value**. The vertical lines show the time of minima and maximum derivative in the PPG pulses in the free finger.

In order to detect the first appearance of the pulses in the finger distal to the cuff (the right hand finger), the extent of each PPG pulse in the left hand finger was digitally determined. The time of the maximal derivative (TD_MX_) was calculated for each pulse in the left finger for subsequent selection of the expected time of the PPG pulses in the right finger (distal to the cuff) in the neighborhood of TD_MX _of the left-finger pulses (see Figure 1). Because the PPG pulse in the finger distal to the cuff is delayed relative to that in the contralateral free finger [13], the maximum derivative in the former was searched for in the region of 100-300 ms after TD_MX _in the latter. The expected PPG pulses in the right finger were searched for by their maximum derivative and not through their minimum, since the slope of the PPG pulse in the neighborhood of the minimum is small, and even low noise can influence the location of the pulse minimum time.

**Figure 2:**
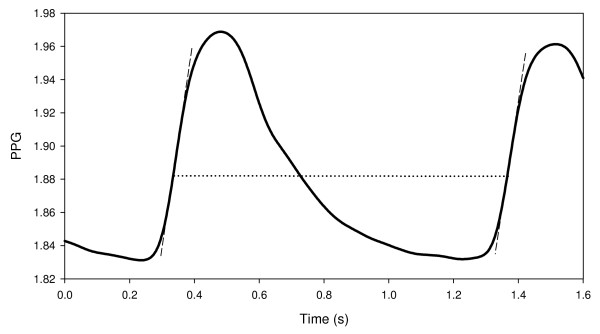
**A PPG pulse demonstrating the point of maximum derivative, and the line connecting the latter with the point of maximum derivative in the next pulse**.

Thus the light transmission curve in the right finger was divided into time segments, from maximum derivative to maximum derivative, each suspected of containing a full PPG pulse (say, from maximum derivative of a PPG pulse to maximum derivative of the next PPG pulse). In order to determine whether the signal in a given segment is noise or a PPG pulse, two parameters were calculated in each segment: an area parameter, which is related to the pulse waveform and the cross-correlation of the signal in each segment with the signal in the neighboring segments. A signal in a given segment was identified as potential PPG pulse, if its pulse waveform was similar to that of a PPG pulse and it correlated with the signal in the neighboring segments. Both the pulse waveform and the cross-correlation parameters were calculated for each pulse after detrending: the line connecting the two points of the maximum derivative in each time segment was subtracted from the signal curve (see Figure 2). The details of the analysis and the criteria for the determination of the appearance of the first PPG pulse are described in Appendix 1.

## Results

The difference between the sphygmomanometric SBP values measured by the two observers was generally less than 4 mmHg. In four cases, where the difference was higher than 4 mmHg, the examinations were repeated. The range of SBP value, measured by sphygmomanometry (SBP_S_) was 91.5-188.5 mmHg.

**Figure 3:**
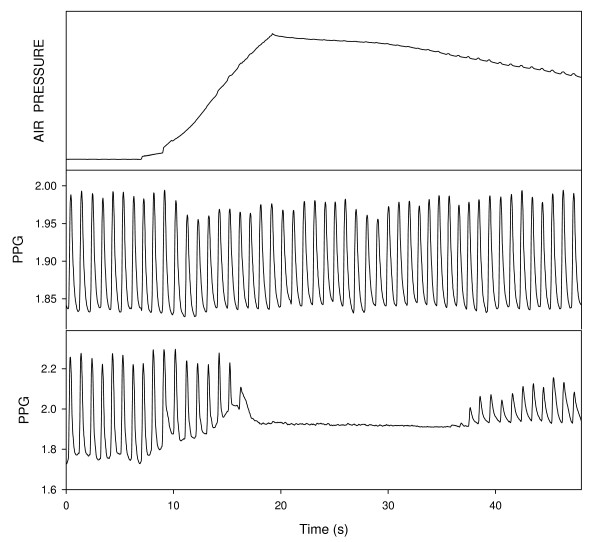
**The curves of air pressure in the cuff (above) and the PPG signals, in the finger distal to the cuff (below) and in the contralateral finger (middle) as function of time**. The PPG signal disappears for air pressure above the systolic blood pressure. Note the oscillometric air-pressure fluctuations in the air-pressure curve.

During the increase of the cuff-pressure the PPG pulses disappeared, when the cuff pressure was sufficiently high, and they reappeared when the cuff-pressure sufficiently decreased. Figure 3 shows the curves of the PPG in the two hands as a function of time together with the cuff pressure measurement during cuff inflation and deflation. The PPG pulses in the finger distal to the cuff disappeared when the cuff pressure increased to above SBP value and reappeared when the cuff pressure decreases to below SBP value, while the PPG pulses in the contralateral finger showed only a minor change. In the majority of the examinations the reappearance of the pulses was easily determined, but in some cases the small PPG pulses in the neighborhood of SBP were not clearly demonstrated above the noise.

**Figure 4:**
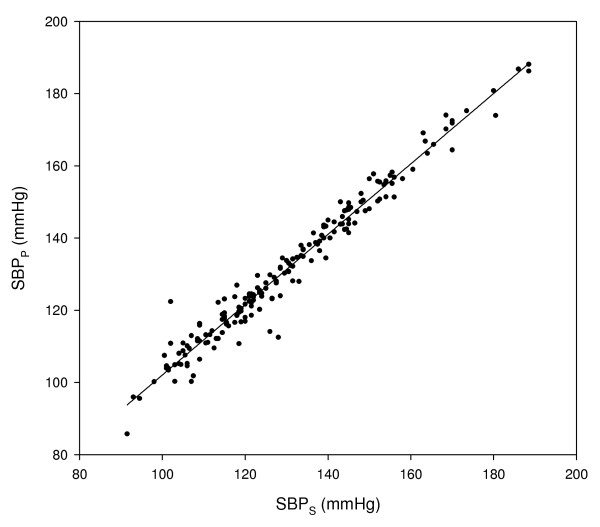
**SBP measured by PPG, SBP_P_, as a function of SBP measured by sphygmomanometry, SBP_S_**.

Figure 4 presents SBP value, as measured by the automatic PPG method (SBP_P_), as a function of SBP_S_. The correlation coefficient between SBP_P _and SBP_S _was 0.983.

**Figure 5:**
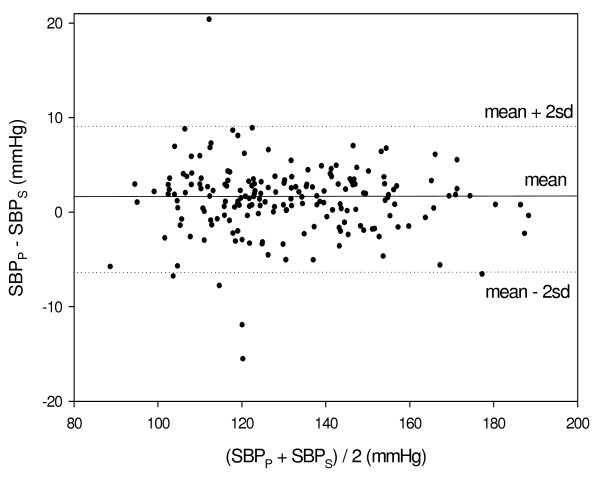
**Bland-Altman plot presenting the values of the difference SBP_P _- SBP_S _as a function of the mean of SBP_S _and SBP_P_**. The difference is higher for the lower blood pressure values.

The difference between SBP_P _and SBP_S _(SBP_P _- SBP_S_) was calculated for each examination, and the mean and SD of these differences were 1.3 ± 3.7 mmHg. The mean value of the difference is significantly higher than zero (p < 0.001 in Student t-test). Figure 5 presents SBP_P _- SBP_S _as a function of the mean of SBP_S _and SBP_P_. The deviations between SBP_P _and SBP_S _are higher for lower values of SBP: the SD value of SBP_P _- SBP_S _for the 101 examinations of SBP_S _with values lower than 130 mmHg was 4.3 mmHg and for the 85 examinations of SBP_S _with values higher than 130 mmHg was 2.9 mmHg. The difference between the values of SD for the two groups is statistically significant (p < 0.01 in one-tailed F-test for comparison of variances).

**Figure 6:**
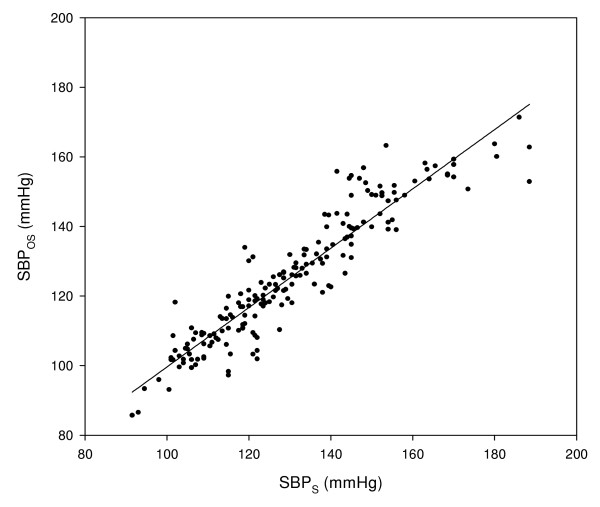
**SBP measured by oscillometry, SBP_OS_, as a function of SBP measured by sphygmomanometry, SBP_S_**.

**Figure 7:**
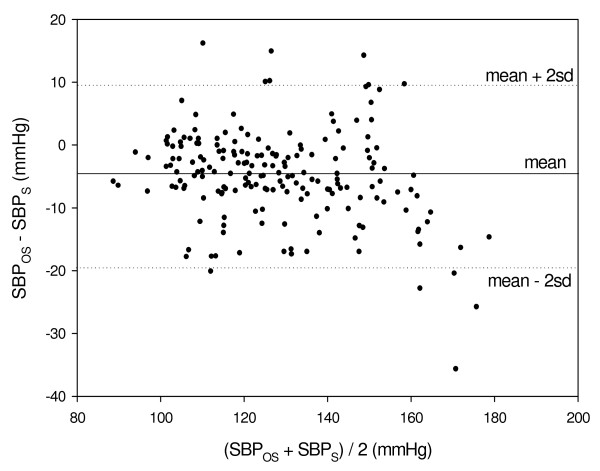
**Bland-Altman plot presenting the values of the difference SBP_OS _- SBP_S _as a function of the mean of SBP_S _and SBP_OS_**.

Figure 6 presents SBP_OS _(SBP value, as measured by oscillometry) as a function of SBP_S_. Figure 7 presents the difference between SBP_OS _and SBPs (SBP_OS _- SBP_S_) as a function of the mean of SBP_S _and SBP_OS_. The correlation coefficient between SBP_OS _and SBP_S _was 0.934, but this value of the correlation coefficient was not sufficient for rendering low value for the standard deviation of SBP_OS _- SBP_S_: the mean and SD of the SBP_OS _- SBP_S _were -4.9 ± 7.3 mmHg.

## Discussion

The disappearance of the Doppler ultrasound and PPG pulses due to the collapse of the artery under the cuff for cuff pressure above SBP value has already been suggested for SBP measurement. In the current study, a technique for automatic PPG-based measurement of SBP is introduced and validated. Our technique is based on three elements: slow increase of the air-pressure in the cuff to above SBP value, simultaneous measurement of the PPG signal in both hands and a suitable algorithm for the detection of the PPG signal when the pressure in the cuff decreases below SBP value.

The algorithm for the automatic detection of the reappearance of the PPG signals in the finger distal to the cuff was found to be efficient, despite the small signal-to-noise ratio in some examinations. Comparison of the automatic technique to sphygmomanometry, the reference standard, shows that the SD of their difference was low, relative to the maximal value required by the standards for automatic blood pressure meters imposed by AAMI.

The advantage of the PPG-based technique over sphygmomanometry is that the former can be done automatically, enabling accurate SBP measurements at home and objective accurate SBP measurements at the physician office. The PPG-based technique is based on direct detection of the opening of the arteries under the cuff, in contrast to oscillometry, which is based on empirical criteria (air-pressure oscillations in the cuff appear even for cuff pressure which is above SBP value). The potential of the PPG-based technique for providing accurate SBP measurement is therefore greater than that of oscillometry. In comparison with sphygmomanometry we obtained SD value of 3.7 mmHg for the PPG-based technique while for oscillometry we obtained SD value of 7.3 mmHg, which is comparable to the maximal SD (8 mmHg) allowed in AAMI standards for automatic blood pressure meters. Note, however, that we used a very simple algorithm for the determination of SBP from the oscillometry curve, while commercial devices which are based on oscillometry, probably use more sophisticated algorithms. On the other hand the presented PPG-based technique is yet preliminary and it can still be improved.

The main limitation of the PPG-based technique is that it can only assess the SBP and it does not allow diastolic blood pressure measurement. The latter can be obtained from the PPG-based device through oscillometry, with its relative low accuracy. It should be noted that mere measurement of SBP also has clinical significance. Subjects with isolated systolic hypertension or even borderline isolated systolic hypertension are at increased risk of developing cardiovascular diseases [14-17] and treatment of isolated systolic hypertension in older adults results in reduced cardiovascular event rates [14,17,18]. An automatic technique for accurate measurement of SBP is therefore of significant clinical merit.

Another disadvantage of the current technique is the need for two PPG probes on the two hands: the second PPG probe in the free hand is used for the determination of the time of the appearance of the potential PPG signal in the finger distal to the cuff. This time of appearance can also be derived from the air pressure pulses in the cuff, so that the second PPG probe can be avoided. In a preliminary study we have found that this technique can be implemented, but its accuracy is lower than that of two PPG probes. Further work is required for the development of accurate technique for automatic SBP measurement based on a single PPG probe.

Further work is also required to examine the validity of the technique for subjects affected by peripheral vasoconstriction, such as during exposure to low temperature environment. In general, low room temperature decreases the PPG signal and consequently decreases the accuracy of the PPG-based technique.

A limitation of the current study is that the PPG-based technique was measured on only male subjects. The parameters of the algorithm for female subjects may be different and the accuracy of the technique may be lower because the PPG signal in females is generally smaller than that in males. An algorithm suitable to females has to be developed, and then a study which compares the results of the technique for males and females can be performed.

## Competing interests

MN tried to commercialize the technique in a start-up company, Ninbar, and still holds shares in it. Ninbar failed to raise sufficient investment capital, so the company is not functioning at present. Two US patents for the technique were assigned to the Jerusalem College of Technology, with MN as the inventor.

## Authors' contributions

MN invented the technique, designed the study, analyzed the data and wrote the paper; AP was responsible for developing the algorithm for the technique; ZG and ATW were responsible for the examinations and contributed to the analysis of the results.

All authors read and approved the final manuscript.

## Appendix 1

As explained in the Materials and Method section, the PPG signal in the right finger was divided into time segments, from maximum derivative to maximum derivative. In order to determine whether the signal in a given segment is a PPG pulse, two parameters were calculated in each segment: an area parameter, which is related to the pulse waveform and cross-correlation of the signal in each segment with the signal in the neighboring segments.

The area parameter (PF - pulse waveform) was calculated from the integral of the signal after detrending for the first half (INT1) and for the second half (INT2) of the segment. For normal PPG pulse INT1 is positive and INT2 is negative so that (INT1-INT2) is positive. If the signal is composed of arbitrary noise mainly containing frequencies higher than 1 Hz, PF is expected to be small. (INT1-INT2) was taken as the pulse pattern parameter PF.

The correlation coefficient (CC) was calculated for each segment between the signal in the segment and the signal in each of the two neighboring segments. If two segments were not equal the longer one was shortened by eliminating the required number of samples. The final value of the correlation coefficient of the segment was taken as the higher of the two values of the correlation coefficient. If CC was higher than a predetermined value the signal was considered as PPG pulse. In preliminary examinations we have found that better results were obtained when the borders of the segments of the PPG pulses in the *right finger *for calculating the correlation coefficient were taken as the time-points of maximum derivative TD_MX _of the corresponding PPG pulses of the *left finger*. Thus the borders of the segments for CC calculation were determined by TD_MX _values in the left finger and the borders of the segments for PF calculation were determined by TD_MX _values in the right finger.

The values of PF and CC for each pulse were used for the determination of the appearance of the first PPG pulse during the cuff deflation. *Based on former examinations *the first PPG pulse was taken as the first of seven consecutive segments, which complied with one of the following two conditions, obtained empirically:

1. At least five segments had CC value higher than 0.85, and PF value higher than 1% of P_I_, the average value of PF for the distal finger before the inflation.

2. At least five segments had CC value higher than 0.65, and for three of them PF value was higher than 7% of P_I_. For the other two segments PF value was higher than 10% of P_I_.

The SBP value was the value of the cuff pressure corresponding to the first of the seven consecutive segments.
